# Validation of revision data for total hip and knee replacements undertaken at a high volume orthopaedic centre against data held on the National Joint Registry

**DOI:** 10.1186/s13018-019-1304-9

**Published:** 2019-10-10

**Authors:** Irrum Afzal, Sarkhell Radha, Tomislav Smoljanović, Giles H. Stafford, Roy Twyman, Richard E. Field

**Affiliations:** 1 South West London Elective Orthopaedic Centre, London, KT18 7EG UK; 2 Imperial College London, South Kensington, London, SW7 2AZ UK

**Keywords:** Arthroplasty, Hip arthroplasty, Knee arthroplasty, Revision arthroplasty, National Joint Registry, Joint registry data, Arthroplasty registries

## Abstract

**Background:**

With over 2.35 million records, the National Joint Registry (NJR) is the largest arthroplasty registry in the world. It provides a powerful tool to monitor implant survivorship and influence different surgical strategies. To date, little work has been undertaken to investigate the validity of the ‘*Reason for Revision*’ recorded in Consultant Outcome Reports on the NJR.

**Methods:**

The NJR was queried to identify all revisions on the THR performed at a single centre over an 11-year period. Review and validation of ‘*Reason for Revision*’ for each case was undertaken using radiological imaging studies, pathology, histology, microbiology and electronic medical records.

**Results:**

Of the 22,046 primary total hip replacements (THR) and total knee replacements (TKR) undertaken by 23 surgeons at our hospital, over an 11-year period, 1.35% (297) were subsequently reported to the NJR as revised. Discrepancies in reporting to the NJR were identified for 41 cases (25.63%) for THR and 28 (20.40%) cases for TKR. Revision for infection was under-reported for both THR and TKR by 1.88% and 3.65% respectively. Reporting of adverse soft tissue reaction to particulate debris for THR was unreported by 11%. Progressive arthritis following a TKR was unreported by 6.56%. All the cases reported as ‘other’ (8.75% for THRs and 3.65% for TKRs) were reclassified to the most appropriate ‘reason for revision’ category. The ‘reason for revision’ data is recorded to the NJR with findings at the time of surgery. It is some days before microbiology and histology reports become available and source data is not always updated.

**Conclusion:**

If an average of 23% wrong data entry at a highly organised institution is replicated throughout the UK, a formal process to validate primary and revision data submitted to the NJR should be considered. Local scrutiny, review and validation of revision data are all vital to optimise the value of the NJR. Accurate data recorded to the NJR is imperative to provide safe and effective improvements in orthopaedic surgery.

## Introduction

Implant registries began over 40 years ago with the Swedish Knee Register in 1975 followed by the Swedish Hip Register in 1979. In 2016, the International Society of Arthroplasty Registries (ISAR) reported 31 arthroplasty registers around the world and ten separate in the USA [1]. The purpose of joint registries is to monitor the performance of arthroplasties and the effectiveness of different types of surgery [2]. The National Joint Registry (NJR) was set up by the Department of Health and Welsh Government in 2002 to collect information on all ankle, elbow, hip, knee and shoulder replacement operations. It has now been mandated that all cases of primary and revision total hip and knee replacement (THR and TKR) procedures, undertaken in England and Wales, are recorded onto the NJR [3].

Annual reports generated using the data from arthroplasty registries are increasingly cited in the rationale for aspects of implant selection, component fixation and other variables of surgical practice. One of the outputs of the data recorded in the NJR is Surgeon (Consultant) Reports. These are provided directly to the individual consultants detailing their annual and 3-year activity. The reports include information on the number of operations undertaken by the consultant in the preceding 1- and 3-year periods, the relative frequency of the different implant fixation techniques used by the consultant, and the relative frequency of implants with different Orthopaedic Data Evaluation Panels (ODEP) ratings. The report also details the consultants’ primary joint replacements, which have subsequently gone on to being revised. The NJR defines ‘revision’ as ‘an operation to remove or replace one or more components of a joint prosthesis’ [3]. In addition, the 90-day mortality for the consultant’s patients is detailed. With such influence on clinical practice, validation of the data reported to the NJR is critical, if the NJR is to be used for feedback and recommendations to the public, hospitals and clinicians and researchers.

In this study, we have reviewed a cohort of patients whose primary hip or knee replacement was undertaken at our centre and used the NJR Consultant Level reports of our surgeons to identify which of the cases have been revised. We then used a combination of data sources to analyse whether the NJR had been provided with the correct ‘reason for revision’ for each procedure.

## Methods

In order to identify the revision cases that had been reported to the NJR for operations where the primary was undertaken at our adult elective orthopaedic centre, we reviewed the NJR Consultant Level reports from the consultants working at this multi-surgeon orthopaedic centre in London. An inclusion and exclusion criteria for data collection for the consultant NJR was set. All patients listed in the revision section of the individual consultant NJR where the primary was undertaken at our centre were included and no patients were excluded.

### Inclusion criteria

Surgeons who were currently working at the centre and had recorded activity on the NJR from1 January 2004 until 31 March 2015

### Exclusion criteria

1. Surgeons who had left the centre
2. Surgeons who were working at the centre at the time of study but did not have recorded NJR activity until 31 March 2015 (newly arrived surgeons)
3. Surgeons who failed to provide informed consent to their NJR Consultant Level reports

A total of 23 consultants out of the 36 consultants practising at this centre met the inclusion criteria and were contacted. Informed consent was obtained prior to viewing the Confidential Consultant NJR reports. Acceptance and informed consent was 95.65% for THR data and 100% for TKR data. Only one surgeon declined to provide access to their NJR THR data; therefore, their THR data was not analysed and they were excluded from the THR study.

The data from the Consultant NJR reports was validated against electronic patient records on Bluespier™ (Bluespier International, Greenbank House, Galton Way, Hadzor, Droitwich, Worcestershire, WR9 7ER), an in-house Outcomes programme, a picture archiving and communication system (PACS), microbiology, histology and any other relevant clinical documentation.

This study did not require ethical committee approval as it was a service evaluation and only involved retrospective data analysis from data collected on the NJR, Bluespier™ and an in-house Outcomes programme. No patient contact was required and no patient received any additional investigations, appointments, correspondence, treatments or any other contact from the study investigator or consultants.

Data from both primary and revision THRs and TKRs must be submitted to the NJR [3]. There are two methods for this data to be submitted to the NJR. One option is by completing a paper (H1 (primary THR hip form)/(K1 primary TKR knee form) or a H2 (revision THR hip form)/K2 (revision TKR knee form). Once completed, the information on the paper forms is entered by clerical staff and uploaded to the NJR. The other option is a twofold electronic upload to the NJR. Initially, the consultant in charge or a member of their team creates an operation note on our patient electronic record system, Bluespier™. This data is then later bulk uploaded to the NJR. The latter method was used in this study. In both, the paper or the electronic H2 and K2 forms, the ‘indication for surgery’ must be provided at the time of surgery. The bulk upload facility allows a hospital to collect NJR data through their own IT system and then transfer it as a block of multiple data records, at regular intervals, to the NJR database [4]. Bulk upload to the NJR is potentially advantageous as it avoids transcription errors that may occur during manual data input to the NJR. This should preserve data quality and save time for consultants and other health care professionals [4].

Data linkage between the NJR, Bluespier™ and our in-house Outcomes programme was undertaken for the validation exercise. NJR Consultant Level reports, Bluespier™ and our in-house Outcomes programme were linked using the local eight-digit hospital ID.

### Validation

Validation of the NJR revision data was a sequential multi-level matching process using data from the NJR, Bluespier™ and an in-house Outcomes programme (Table 1).

**Table 1:** The fields of validation used in the study to validate the NJR revision data

Field name	Variable type	Comment
Local patient ID	Continuous	
Gender	Dichotomous	Male or female
Date of birth	Continuous	
Revised by selected surgeon	Dichotomous	Yes or no
Side	Dichotomous	Left or right
Date of revision	Continuous	
Date of primary	Continuous	
Time from primary	Continuous	
Primary type	Categorical	Primary cemented, primary uncemented, primary hybrid, primary resurfacing
Reason (indication) for revision NJR	Categorical	

#### Fields of validation

The reason (indication for revision) NJR categories for the revision hip replacement (H2 form) and the revision knee replacement (K2 form) are shown in Table 2.

**Table 2:** The categories for the revision hip replacement form (H2 form) and the revision knee replacement form (K2 form)

Indication for revision—NJR categories for the revision hip replacement form (H2 form)	Indication for revision—NJR categories for the revision knee replacement form (K2 form)
Adverse soft tissue reaction to particulate debris	Aseptic loosening
Aseptic loosening	Component dissociation
Dislocation/subluxation	Dislocation/subluxation
Dissociation of the liner	Implant fracture
Head-socket mismatch	Infection
Implant fracture	Instability
Infection	Lysis
Lysis	Malalignment
Malalignment	Other
Other	Peri-prosthetic fracture
Peri-prosthetic fracture	Progressive arthritis to the remaining knee
Unexplained pain	Stiffness
Wear of acetabular component	Unexplained pain
	Wear of the polyethylene component

We introduced two new categories through our validation process for the THR revisions, squeaking and failed osseointegration. As an exercise, we subdivided the cases from aseptic loosening to failed osseointegration and aseptic loosening. The cases allocated to failed osseointegration were the primary THRs with no radiological evidence of osseointegration within 3 years of implantation.

Each revision case was retrospectively reviewed under the supervision of two senior hip and knee consultants, and their agreement was evaluated. For each case, the clinical information, operative information, microbiology and imaging (x-rays, CT and MRI scans) and any other relevant clinic documentation from Bluespier™ or our in-house Outcomes programme was reviewed to determine whether the indication for each revision recorded to the NJR was correct.

## Results

From 1 January 2004 until 31 March 2015, a total of 37,014 primary hip and knee replacements were recorded as having been undertaken at our centre. These comprised 16,931 THRs and 20,083 TKRs. Of the 37,014 operations, 22,046 were included in this study. These comprised of 9411 THRs undertaken by 22 surgeons and 12,635 TKRs undertaken by 23 surgeons.

1.70% (160) of these primary THRs and 1.08% (137) of these primary TKRs were reported to the NJR as revised either by the consultant who undertook the primary procedure or by another consultant either at our centre or at another hospital (Table 3).

**Table 3:** A breakdown of the results for both total hip replacements and total knee replacements

	Total hip replacement (THR)	Total knee replacement (TKR)
Time period	01 January 2004 to 31 March 2015	01 January 2004 to 31 March 2015
Total of number of primaries undertaken at our centre	16,931	20, 083
Number of surgeons involved in this study	22	23
Number of primaries undertaken at our centre by the surgeons involved	9411	12,635
Number of revisions reported to the NJR where the primary THR or TKR was undertaken at our centre by the surgeons involved in the study	160	137
Percentage of revisions where the primary THR or TKR was undertaken at our centre	1.70%	1.08%

This study revealed a 25.63% and 20.4% error rate in the indication for revision between what is reported to the NJR and the findings post-validation at our centre for THRs and TKRs respectively (Table 4).

**Table 4:** The number and percentage of discrepancies in reporting to the NJR

	Total hip replacement (THR)	Total knee replacement (TKR)
Number of revisions reported to the NJR where the primary THR or TKR was undertaken at our centre by the surgeons involved in the study	160	137
Discrepancies in reporting reason (indication) for revision NJR	41	28
Percentage of discrepancies (%)	25.63%	20.40%

The key discrepancies for THRs were in the reporting of adverse soft tissue reaction to particulate debris, infection, aseptic loosening, and ‘other’. Adverse soft tissue reaction to particulate debris was under-reported to the NJR by 11%. The ‘reason for revision’ data is recorded to the NJR with findings at the time of surgery. It is some weeks before histological evidence becomes available and the source data is not updated. Likewise, infection was under-reported to the NJR by 1.88%. Microbiology reports for infection are also not immediately available. Aseptic loosening was over-reported by 5.60%, and these cases were reclassified to either adverse soft tissue reaction to particulate debris or failed osseointegration.

Discrepancies in reporting to the NJR were identified for 28 (20.4%) cases for TKRs. The most frequent discrepancies were in the reporting of infection, progressive arthritis, malalignment and ‘other’. Progressive arthritis and malalignment were under-reported by 6.56% and 3.65% respectively. Infection was under-reported to the NJR by 3.65%. 8.75% and 3.65% of the revised cases were reported as ‘other’ for THR and TKR respectively. Retrospective review allowed all these cases to be reclassified to the most appropriate ‘reason for revision’ category.

There were no reported discrepancies in the fields of validation (local patient ID, gender, date of birth, revised by selected surgeon, side, date of revision, date of primary, time from primary and type of primary) for both THRs and TKRs.

## Discussion

Our data indicated discrepancies of 25.6% and 20.4% for THRs and TKRs respectively. The results obtained in this study are from a broad spectrum of patients with degenerative hip and knee disease over an 11-year period. Data from the NJR is reliant on the accuracy of the data entered into it from the hospital by the surgeon, consultant in charge or the health care team. The bulk upload process is taking the data from the operative note and is a direct duplication to the NJR by an IT system. The other mechanism of paper forms can be data entered wrong by the data entry clerks. The NJR takes the data from the bulk upload at face value and does not validate it. In this study, the data is taken from the H2 and K2 form completed online on a form, which is a direct replication of the paper form. The form is completed by the operating surgeon, consultant surgeon or a member of the health care team.

Validating the NJR data is fundamental as the registry holds over 2.35 million patient records. It is crucial to know that the data is accurate as the data is used on a patient, surgeon, hospital, the Department of Health and international level. Any evaluation or interpretation of registry data is dependent upon the veracity of the dataset. If major discrepancies are identified, hospitals, surgeons and allied health professionals should report back to the NJR. In addition, other hospitals can be notified to complete audits in order to check the accuracy of their data.

To best of our knowledge, no other papers have been published to show their validity of the ‘reason for revision’ in the Consultant NJR reports in the NJR. Therefore, a study from a high-volume, multi-disciplinary elective orthopaedic centre with access to patient medical records, laboratory information and imaging information is required to gain more accurate information on the ‘reason for revision’. This study was able to fulfil this aim.

Our discrepancies correlate with previous studies in the literature. A team at the London Implant Retrieval Centre (LIRC) in Stanmore have validated primary metal-on-metal (MoM) hip arthroplasties for England, Wales, and Northern Ireland using a NJR dataset [5]. From their data analysis, they were unable to link 39.1% of their primary procedures to the NJR. They also identified a high error rate (16.6%) for outcome coding on the NJR [6]. In a study from validating the infections, *Validation of the diagnosis ‘prosthetic joint infection’ (PJI) in the Danish Hip Arthroplasty Register (DHR)*, only two thirds of revisions for PJI were captured in the DHR, and only 77% of the diagnoses of PJI reported to the register could be confirmed [7]. Another study from New Zealand underestimated the rate of reoperation for PJI by one third [8].

Data from the NJR is reliant on the accuracy of the data entered into it from the hospital by the surgeon, consultant in charge or the health care team. This study identified the importance of retrospectively checking the data provided to the NJR. This study identified the importance of local hospital-based databases, which consolidate information and knowledge on revision arthroplasty. A previous study looking at the NJR anaesthetic data also highlighted the importance of data following their review in anaesthetic discrepancies reported on the NJR [9].

We recommend that if greater than 20% wrong data entry at a highly organised institution is replicated throughout the UK, a formal process to validate the revision data provided to the NJR should be considered.

In order to improve both arthroplasty and NJR data quality, it is crucial that accurate data is recorded to the NJR. The surgeons and consultants in charge should take ownership in ensuring all procedures are accurately recorded. It is important that surgeons undertake independent evaluations of their operative data and the data held on the NJR. It is also important for hospitals to undertake the same exercise, and if discrepancies are identified, the NJR should be notified at the earliest opportunity.

The strength of this study is this study was undertaken using data from a highly organised centre dedicated to elective orthopaedic work. However, there are limitations and this may be an underestimate, as it cannot be guaranteed that all revision procedures have been reported to the NJR.

For THRs, our data in this study pre-dates 2012. In 2012, it was mandated by the NJR to report adverse soft tissue reaction to particulate debris following a primary THR as a revision procedure. Therefore, a limitation of this study is surgeons performing a revision for adverse soft tissue reaction to particulate debris would not have had the option to select adverse soft tissue reaction to particulate debris; as a result, this correlates to the under-reporting of adverse of soft tissue reaction.

## Conclusion

In conclusion, our study is the first of its kind to individually review a subset of revision cases submitted to the NJR. As we have identified the discrepancies of 25.6% (41) and 20.4% (28) for THRs and TKRs respectively, we recommend local scrutiny, review and validation through a multi-disciplinary team (MDT) across centres involved in revision arthroplasty. An effective pathway to review all cases following a revision THR and TKR should be implemented in every centre. The ‘indication for revision’ provided to the NJR should be confirmed, and the NJR should immediately be informed and updated if any discrepancies are identified.

## Data Availability

The data that support the findings of this study are available from the NJR and available to the consultant orthopaedic surgeons. Restrictions apply to the availability of these data. Informed consent was obtained from the consultant orthopaedic surgeons to use data in this research. The data for this study is available from the authors upon reasonable request and with permission of the NJR, consultant orthopaedic surgeons and first author.

## Abbreviations

ISAR: International Society of Arthroplasty Registries
MoM: Metal-on-metal
NJR: The National Joint Registry
ODEP: Orthopaedic Data Evaluation Panel
PACS: Picture archiving and communication system
PJI: Prosthetic joint infection
THR: Total hip replacement
TKR: Total knee replacement
